# Home-based cardiac rehabilitation: A review of bibliometric studies and visual analysis of CiteSpace (2012–2021)

**DOI:** 10.1097/MD.0000000000031788

**Published:** 2022-12-09

**Authors:** Jingyu Liu, Lingyu Wang, Haiyan Fang, Xiang Wang, Lingsha Wu, Jing Zhang

**Affiliations:** a College of Nursing, Anhui University of Chinese Medicine, Hefei, China.

**Keywords:** bibliometrics, CiteSpace, home-based cardiac rehabilitation, research hotspots, visualization analysis

## Abstract

Home-based cardiac rehabilitation has been a major area in cardiac rehabilitation research for a long time. However, there are few systematic studies in this field using bibliometric analysis. We collected articles and reviews for home-based cardiac rehabilitation from the Web of Science Core Collection. Our objectives were to perform a bibliometric analysis and visualization study to determine hotspots and trends of home-based cardiac rehabilitation, identify collaboration and influence among authors, countries, institutions, and journals, and assess the knowledge base to develop clinical research in the future. This study will provide a valuable reference for researchers concerned with HBCR.

## 1. Introduction

Cardiovascular disease remains the leading cause of death worldwide today.^[1,2]^ Cardiac rehabilitation is recommended by international guidelines as Level 1A evidence for cardiovascular patients as a comprehensive intervention that integrates exercise training, health education and cognitive behavior change to reduce mortality, readmission rates and improve health-related quality of life.^[3–5]^ However, center-based cardiac rehabilitation (CBCR) has long been underutilized, with studies indicating that only 14% to 35% of heart attack survivors and 31% of post-coronary surgery patients enrolled in CBCR programs among eligible patients.^[6,7]^ Lack of health insurance coverage, commuting barriers, low referral rates, and patients’ own lack of health literacy are the main reasons for low CBCR participation and completion rates. Lack of health insurance coverage, commuting barriers, low referral rates, and patients’ own lack of health literacy are the main reasons for low CBCR participation and completion rates. It is clear that new CR delivery strategies are urgently needed in the face of CBCR’s disproportionately low participation and completion rates. Home-based cardiac rehabilitation (HBCR) has received a lot of attention as a possible alternative modality. Unlike center-based cardiac rehabilitation (CR) services (which are provided in medically supervised facilities), HBCR relies on remote instruction and indirect exercise supervision and is provided primarily or entirely outside of traditional center-based settings. It can be implemented in a variety of settings, including the home or other non-clinical settings such as community centers, health clubs, and parks. Conceptually, HBCR could help overcome some of the barriers faced by CBCR programs, including geographic, logistical, and other access-related barriers. Although CBCR staff often recommend home-based exercise training for patients on days when they are not in a CBCR center, “stand-alone” HBCR programs are still in their infancy. Currently, home-based cardiac rehabilitation programs are covered by health insurance in countries such as Australia, Canada and the United Kingdom. The United States also published a scientific statement on “Home-based Cardiac Rehabilitation” in 2019.^[8]^ HBCR offers new options for patients who are unable to participate in CBCR due to distance, work responsibilities, lack of time, and transportation issues.^[9]^ It not only guarantees the accessibility of CR during the COVID-19 pandemic, but also provides better opportunities for patients in rural or other backward areas to participate in CR.^[10,11]^ The latest meta-analysis indicates that HBCR with wearable sensors has the potential to increase the accessibility, adherence, and participation rates in CR interventions, by helping to overcome several barriers that prevent CR participation.^[12]^ Overall, home-based cardiac rehabilitation has a promising future.

At present, many methods systematically evaluate a research field, among which bibliometrics is one of the most used. Literature metrology is an applied mathematics and statistics method used to investigate the discipline of books and other media.^[13]^ It can be used not only for qualitative and quantitative analysis in a study in the field of authors, institutions, countries, and regions, as well as co-cited authors, journals, and references, but it can also help researchers quickly grasp the research hotspots and development trends of a particular field. This cannot be achieved by other methods such as traditional review, meta-analysis, or experimental research. In recent years, bibliometrics analysis has received increasing attention.^[14–18]^ Owing to its powerful analysis and visualization capabilities, bibliometrics is suitable for the evaluation and review of home-based cardiac rehabilitation.

This study was analyzed using the common bibliometric tool CiteSpace, the purpose of this study was to draw knowledge maps through their powerful network cooperative analysis and discuss the research hotspots and development trends of HBCR over the past 10 years by performing the following steps. First, we analyzed the authors, institutions, and countries/regions, co-cited authors, journals, and literature to obtain the most relevant general information and cooperation information in this field. Second, the knowledge structure and hotspot evolution in the research field of HBCR was determined through a burst analysis of keywords and co-citation literature, providing new ideas for clinical research and applications.

## 2. Methods

### 2.1. Data sources and search strategies

The data analyzed in bibliometrics were downloaded from the Web of Science database. This database was selected because it currently contains more than 12,400 authoritative and high-impact academic journals worldwide, and thus is regarded as the most influential database that can provide the comprehensive data information required by bibliometrics software.^[16,19–21]^ We searched the literature through the Web of Science Core Collection (WoSCC). The advanced search entry in the database was set as (“home-based cardiac rehabilitation” or “tele-cardiac rehabilitation” or “mobile health cardiac rehabilitation” or “cardiac tele-rehabilitation” or “electronic cardiac rehabilitation” or “eHealth-CR” or “mHealth-CR”). the considered period went from January 1, 2012, to December 31, 2021, and selected papers and review papers were downloaded on October 10, 2022. Proceedings papers, conference abstracts, book chapters, online publications, editorial material, revisions, bibliographic items, and letters were excluded. Choose “English” as the language type. In addition, literature published in languages other than English was excluded.

### 2.2. Inclusion criteria

**Figure 1.**
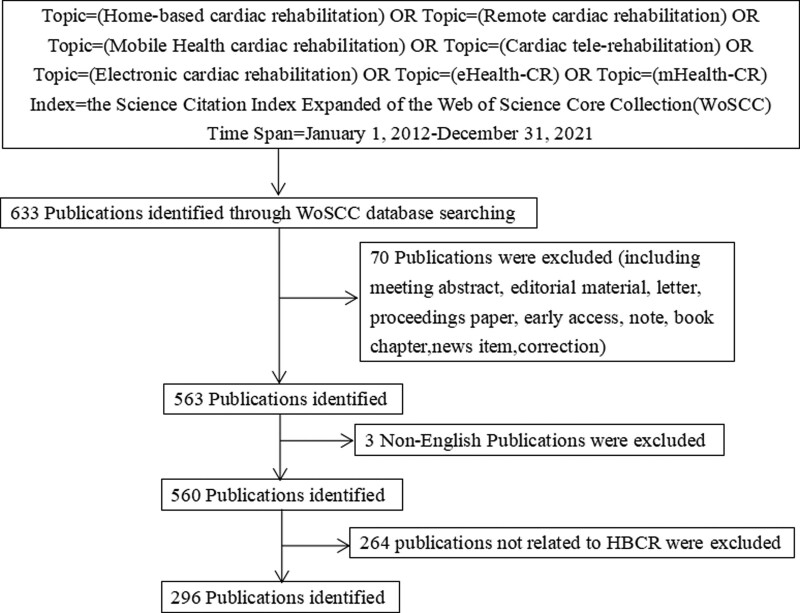
Flow chart of literature screening included in this study.

Referring to other studies,^[22,23]^ all titles and abstracts of the literature were independently reviewed by 2 investigators. After excluding literature not related to home-based cardiac rehabilitation, a total of 296 papers were retained. The literature screening process is shown in Figure 1.

### 2.3. Visualization analysis tool—CiteSpace

This study will use CiteSpace 5.8 R3 software based on JAVA programs for bibliometrics and visual analysis. CiteSpace is an application software for visual analysis of literature, developed by Professor Chaomei Chen’s team at Drexel University, which can support multiple types of bibliometric analysis and research. It includes cooperative network analysis, Keywords co-occurrence analysis, author co-citation analysis, co-citation analysis of reference, etc., which has become a relatively influential analysis software in the field of bibliometrics.^[17]^ We downloaded the records retrieved by WoSCC, and then converted these data into plain text format for export, including complete records and references, (see Supplemental Digital Content, http://links.lww.com/MD/H935), and finally imported into CiteSpace 5.8 R3 for bibliometric and visual analysis.

## 3. Results

### 3.1. Bibliometric analysis of publication years

**Figure 2.**
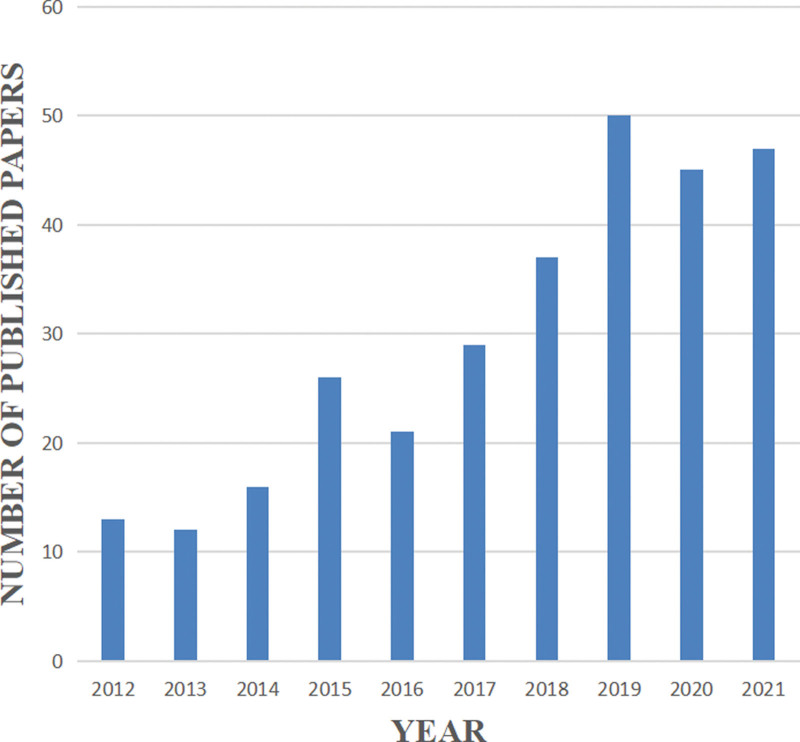
Annual trend chart of publications.

In a specific research field, the number of articles published in each period determines the trend of a certain research hotspot. For the period from 2012 to 2021, we collected a total of 296 eligible articles (Fig. 2). As shown in Figure 2, the number of articles related to home-based cardiac rehabilitation shows an increasing trend, indicating that this research topic has been attracting considerable attention. In particular, the number of articles published reached 50 in 2019.

### 3.2. Bibliometric analysis of authors and co-cited authors

**Figure 3.**
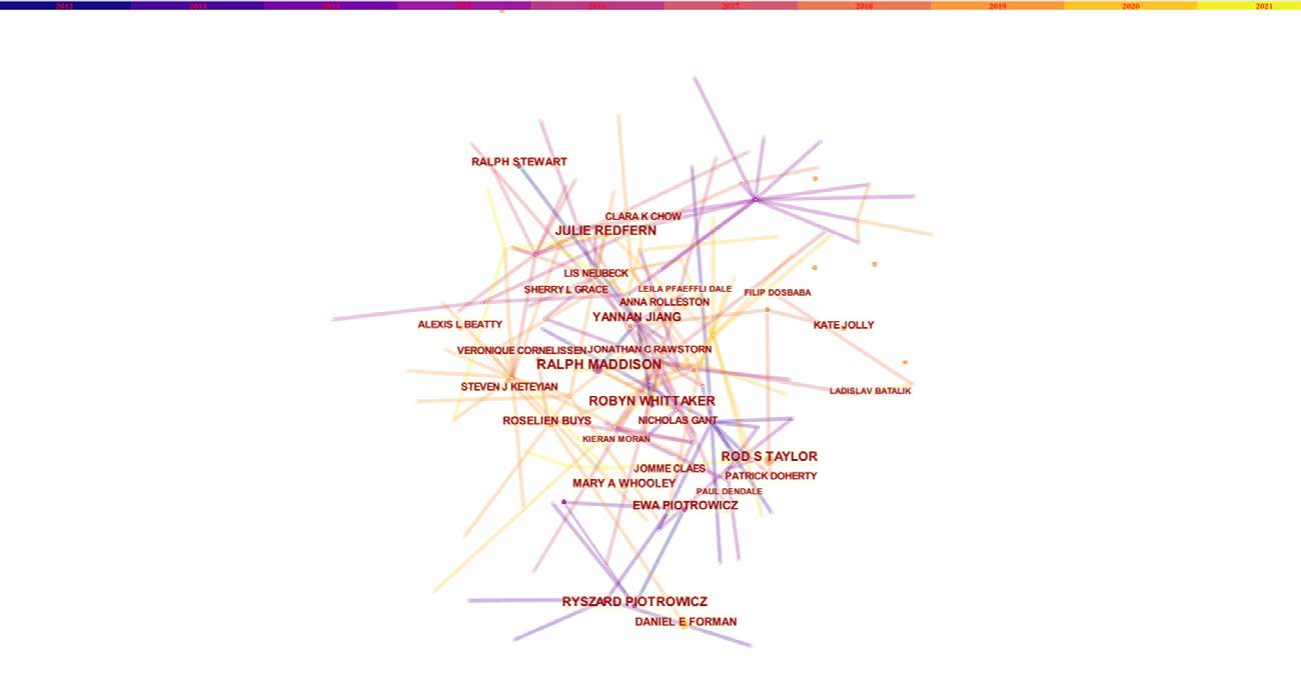
The network of authors.

**Figure 4.**
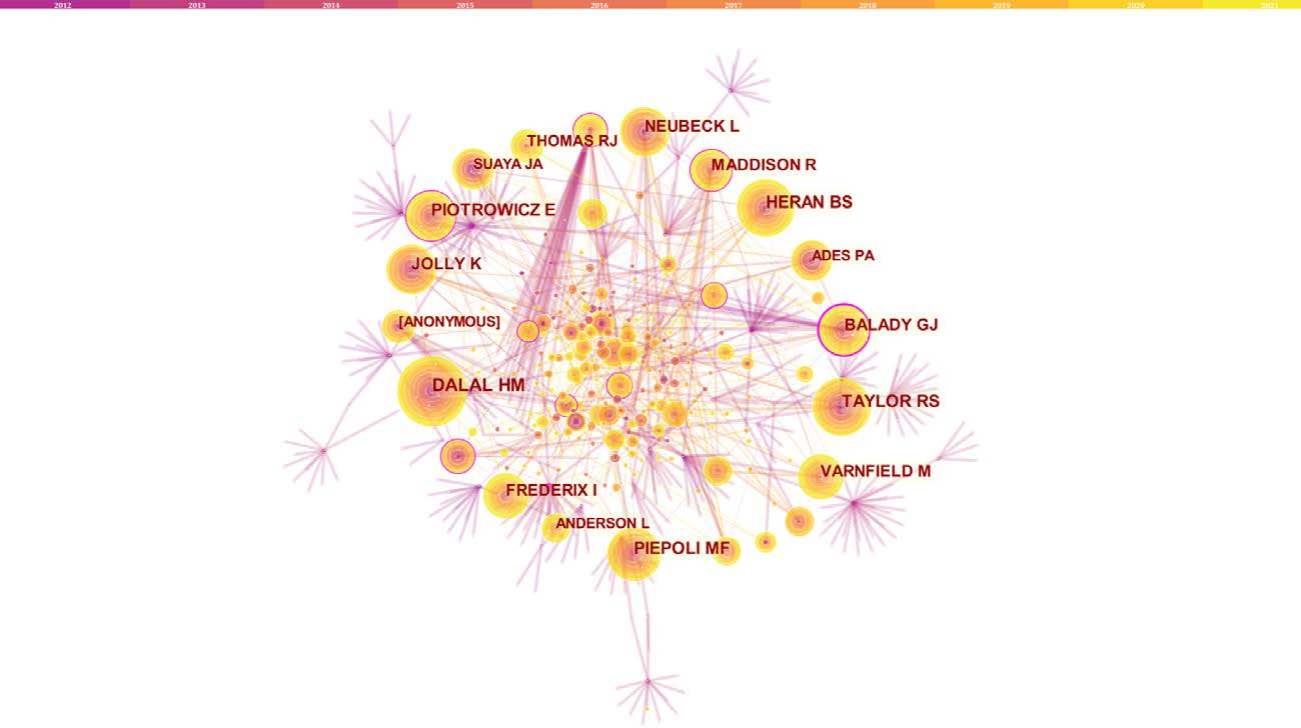
The network of co-cited authors.

In the analysis of authors, a total of 1503 authors were found to have participated in studies related to HBCR, 23 of whom had published more than 5 papers. Ralph Maddison has the highest number of publications (n = 15), followed by Julie Redfern (n = 13), Rod S Taylor (n = 11), Ryszard Piotrowicz (n = 11), and Robyn Whittaker (n = 11) (Table 1). Authors with at least 5 publications (n = 28) are shown in the visualization mapping (Fig. 3). Co-cited authors refer to those who are co-cited in different publications. The results indicated 693 co-cited authors, among which 22 were co-cited more than 30 times. The most frequently co-cited author is Dalal HM (n = 91), followed by Piepoli MF (n = 89), Taylor RS (n = 74), Piotrowicz E (n = 73), Heran BS (n = 70), Varnfield M (n = 63), and Frederix I (n = 60). The remaining authors with co-citation ≥40 (n = 16) are presented on the visualization mapping (Fig. 4). The node size in the figure indicates a high frequency of the co-cited authors.

**Table 1:** The top 10 authors and co-cited authors of HBCR.

Rank	Author	N (%)	Co-cited author	Co-citation	Centrality
1	Ralph Maddison	15 (5.07)	Dalal HM	91	0.04
2	Julie Redfern	13 (4.39)	Piepoli MF	89	0.03
3	Rod S Taylor	11 (3.72)	Taylor RS	74	0.04
4	Ryszard Piotrowicz	11 (3.72)	Piotrowicz E	73	0.17
5	Robyn Whittaker	11 (3.72)	Heran BS	70	0.07
6	Ewa Piotrowicz	10 (3.38)	Varnfield M	63	0.06
7	Yannan Jiang	10 (3.38)	Frederix I	60	0.05
8	Daniel E Forman	8 (2.70)	Balady GJ	59	0.27
9	Ralph Stewart	8 (2.70)	Jolly K	59	0.05
10	Roselien Buys	8 (2.70)	Neubeck L	54	0.06

HBCR = home-based cardiac rehabilitation.

### 3.3. Bibliometric analysis of countries/regions and institutions

**Figure 5.**
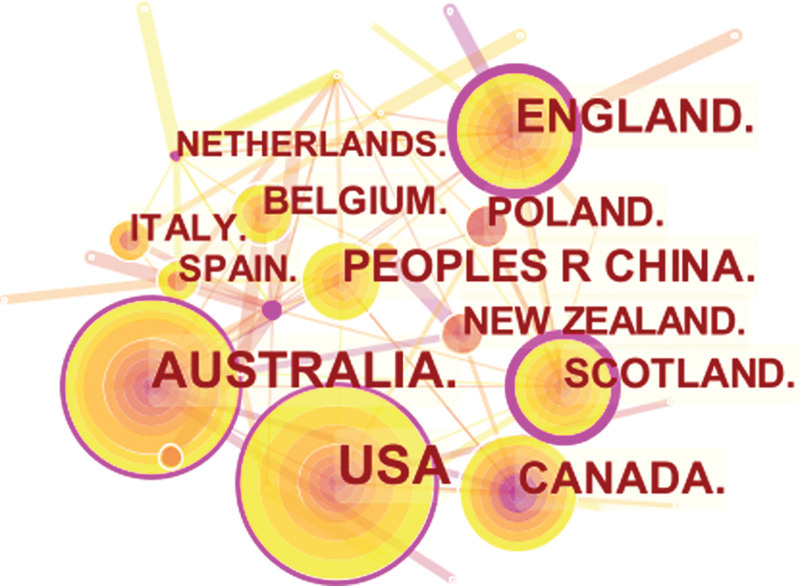
The network of countries.

We used CiteSpace to analyze countries/regions and institutions, and the results showed that a total of 626 institutions from 49 countries/regions jointly published 296 articles. Among the top 10 countries, the US had the largest number of articles (54, 18.24%), followed by Australia (45, 15.20%), England (31, 10.47%), Canada (26, 8.78%), and China (24, 8.11%) (Table 2). Tree ring history represents the record of articles published in a certain country; different colors of the tree ring represent the corresponding time, and the overall size of the tree ring reflects the number of articles published by countries such as the US, Australia, and England, which are characterized by a high centrality (≥0.1). The purple outer ring, shown in Figure 5, is often considered an important turning point leading to a transformative discovery.^[19,24]^ Furthermore, the color of tree rings showed that the Australia (2002), England (2002), Canada (2002), China (2002), Poland (2002), and New Zealand (2003) were the first countries to begin research of HBCR.

**Table 2:** The top 10 countries/regions of HBCR.

Rank	Country/region	N (%)	Year	Centrality
1	USA	54 (18.24)	2013	0.17
2	AUSTRALIA	45 (15.20)	2012	0.15
3	ENGLAND	31 (10.47)	2012	0.34
4	CANADA	26 (8.78)	2012	0.09
5	PEOPLES R CHINA	24 (8.11)	2012	0.06
6	POLAND	20 (6.76)	2012	0.04
7	SCOTLAND	19 (6.42)	2015	0.26
8	BELGIUM	17 (5.74)	2015	0.01
9	NEW ZEALAND	16 (5.41)	2012	0.01
10	SPAIN	13 (4.39)	2013	0.07

HBCR = home-based cardiac rehabilitation.

**Figure 6.**
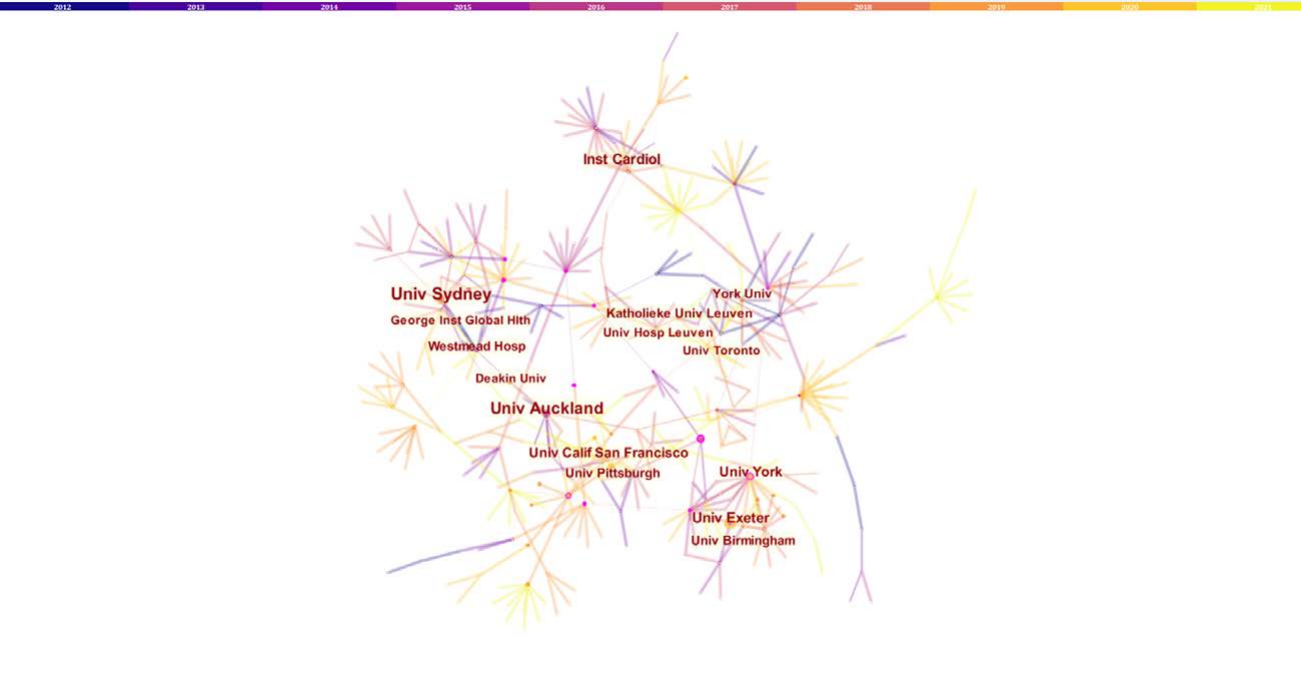
The network of institutions.

Meanwhile, we also observed the institutional collaboration network on the topic of HBCR. The Figure 6 and Table 3 shows that the top 5 institutions were the University of Sydney (18), University of York (17), University of Auckland (16), University of Exeter (12), and Institute of Cardiology (12).

**Table 3:** The top 10 institutions of HBCR.

Rank	Institution	N (%)	Year	Centrality
1	Univ Sydney	18 (6.1)	2012	0.03
2	Univ York	17 (5.7)	2012	0.27
3	Univ Auckland	16 (5.4)	2012	0.06
4	Univ Exeter	12 (4.1)	2015	0.01
5	Inst Cardiol	12 (4.1)	2012	0.03
6	Univ Calif San Francisco	9 (3.0)	2016	0.01
7	Univ Birmingham	9 (3.0)	2015	0.00
8	Westmead Hosp	8 (2.7)	2012	0.15
9	Katholieke Univ Leuven	8 (2.7)	2015	0.02
10	Univ Pittsburgh	8 (2.7)	2019	0.08

HBCR = home-based cardiac rehabilitation.

### 3.4. Bibliometric analysis of co-cited references

Our analysis of the co-cited references found that the top 10 were co-cited more than 238 times, and 3 of these (Varnfield et al, 2014; Maddison et al, 2015; and Chow et al, 2015) were co-cited more than 90 times (Table 4). The most co-cited reference was published by Varnfield et al in 2014,^[25]^ which validated the use and clinical validity of a smartphone-supported CR model for home care in a randomized controlled trial. The second co-cited reference was published by Maddison et al in 2015.^[26]^ The study showed that a mobile phone intervention was effective and probably cost-effective for increasing physical activity and may have the potential to augment existing cardiac rehabilitation services. The third co-cited reference was published by Chow et al in 2015,^[27]^ the study compared various indicators such as mortality and exercise capacity under HBCR and CBCR modes, and found that cardiac rehabilitation effects of the 2 modes were the same in the short term.

**Table 4:** The top 10 co-cited references of HBCR.

Rank	Co-cited reference	Frequency	Centrality
1	Varnfield M, 2014, HEART, V100, P1770, DOI 10.1136/heartjnl-2014-305783	40	0.05
2	Maddison R, 2015, EUR J PREV CARDIOL, V22, P701, DOI 10.1177/2047487314535076	30	0.18
3	Chow CK, 2015, JAMA-J AM MED ASSOC, V314, P1255, DOI 10.1001/jama.2015.10945	26	0.03
4	Rawstorn JC, 2016, HEART, V102, P1183, DOI 10.1136/heartjnl-2015-308966	25	0.08
5	Dale LP, 2015, J MED INTERNET RES, V17, P0, DOI 10.2196/jmir.4944	23	0.08
6	Frederix I, 2015, J TELEMED TELECARE, V21, P45, DOI 10.1177/1357633X14562732	23	0.06
7	Anderson L, 2017, COCHRANE DB SYST REV, V0, P0, DOI 10.1002/14651858.CD007130.pub4	20	0.01
8	Clark RA, 2015, EUR J PREV CARDIOL, V22, P35, DOI 10.1177/2047487313501093	18	0.02
9	Heran BS, 2011, COCHRANE DB SYST REV, V0, P0, DOI 10.1002/14651858.CD001800.pub2	17	0.01
10	Anderson Lindsey, 2017, Cochrane Database Syst Rev, V6, P0, DOI 10.1002/14651858.CD012264.pub2	16	0.11

HBCR = home-based cardiac rehabilitation.

### 3.5. Bibliometric analysis of co-cited journal

**Figure 7.**
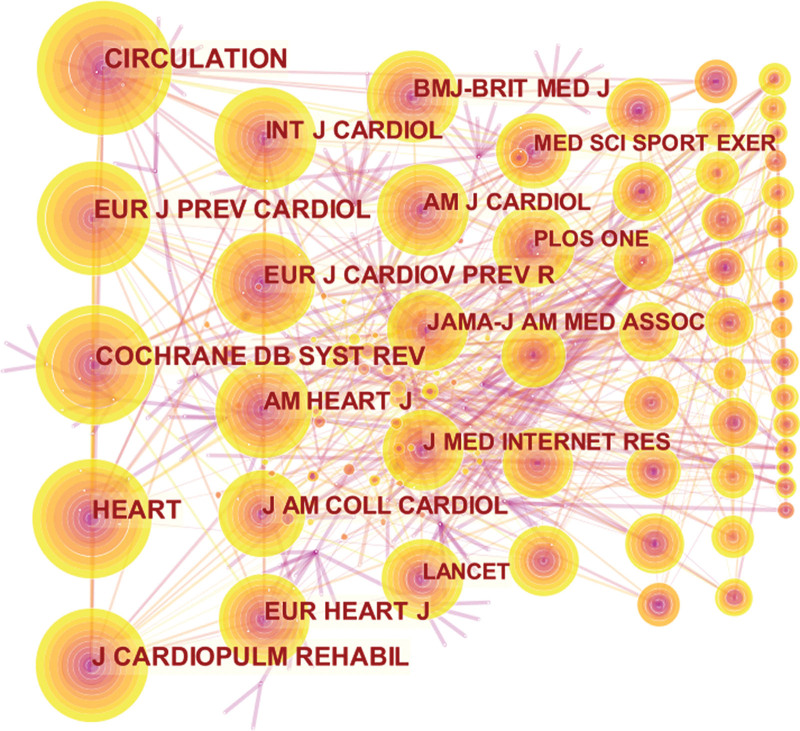
The network of co-cited Journal.

In the analysis of co-cited journals, the results showed that among 364 co-cited journals, 11 journals were co-cited more than 100 times. As shown in Table 5 and Figure 7, the most co-cited journal was Circulation, and the journal with the highest impact factor (IF) value was European Heart Journal. Seven of the top 10 cited journals have an IF value greater than 1, 2 have an IF value greater than 5, and 6 belong to the journal citation reports Q1 division.

**Table 5:** The top 10 co-cited Journals of HBCR.

Rank	Frequency	Journal Name	IF (2021)	JCR
1	203	Circulation	6.47	Q1
2	175	European Journal of Preventive Cardiology	1.32	Q1
3	159	Cochrane Database of Systematic Reviews	1.34	Q1
4	157	Heart	1.44	Q1
5	152	Journal of Cardiopulmonary Rehabilitation and Prevention	0.53	Q2
6	137	International Journal of Cardiology	0.92	Q2
7	132	European Journal of Cardiovascular Prevention & Rehabilitation	–	Q2
8	131	American Heart Journal	1.1	Q2
9	121	Journal of the American College of Cardiology	4.71	Q1
10	113	European Heart Journal	6.77	Q1

HBCR = home-based cardiac rehabilitation, JCR = journal citation reports.

### 3.6. Bibliometric analysis of co-occurring keywords and cluster

**Figure 8.**
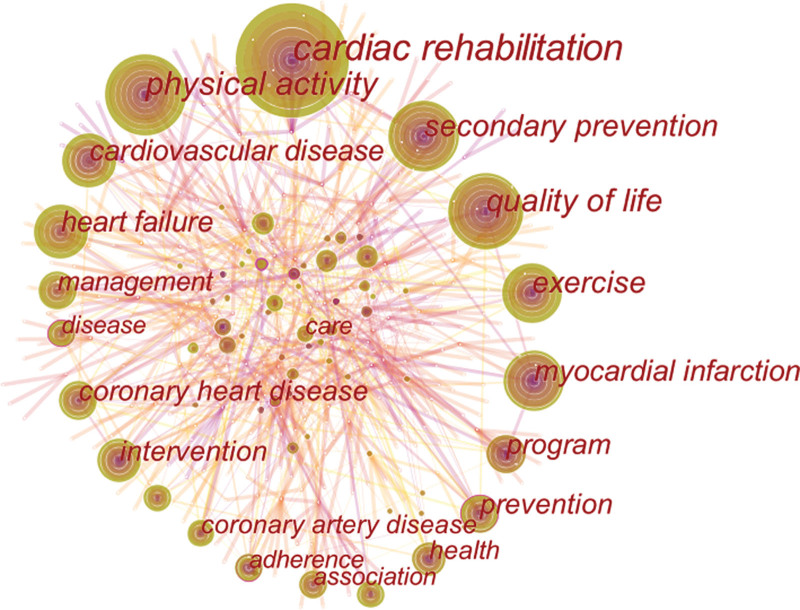
The network map of keywords.

Keyword co-occurrence analysis is the keyword analysis provided by the authors in the dataset. Keyword co-occurrence and emergent items were analyzed by CiteSpace software. A total of 574 keywords were found by CiteSpace. Keywords related to HBCR are presented in the network diagram (Fig. 8). The top 10 keywords cardiac rehabilitation (161), physical activity (85), quality of life (69), secondary prevention (60), exercise (55), cardiovascular disease (53), myocardial infarction (50), heart failure (48), coronary heart disease (44), and intervention (41) (Table 6). Among all the keywords, 37 appeared more than 10 times and 6 appeared more than 50 times.

**Table 6:** The top 10 keywords of HBCR.

Rank	Frequency	Keywords	Centrality	Keywords
1	161	Cardiac rehabilitation	0.20	Participation
2	85	Physical activity	0.16	Scientific statement
3	69	Quality of life	0.13	Trial
4	60	Secondary prevention	0.12	Adherence
5	55	Exercise	0.12	Risk factor
6	53	Cardiovascular disease	0.11	Prevention
7	50	Myocardial infarction	0.11	Disease
8	48	Heart failure	0.11	Validity
9	44	Coronary heart disease	0.11	Update
10	41	Intervention	0.10	Coronary heart disease

HBCR = home-based cardiac rehabilitation, IF = impact factor.

**Figure 9.**
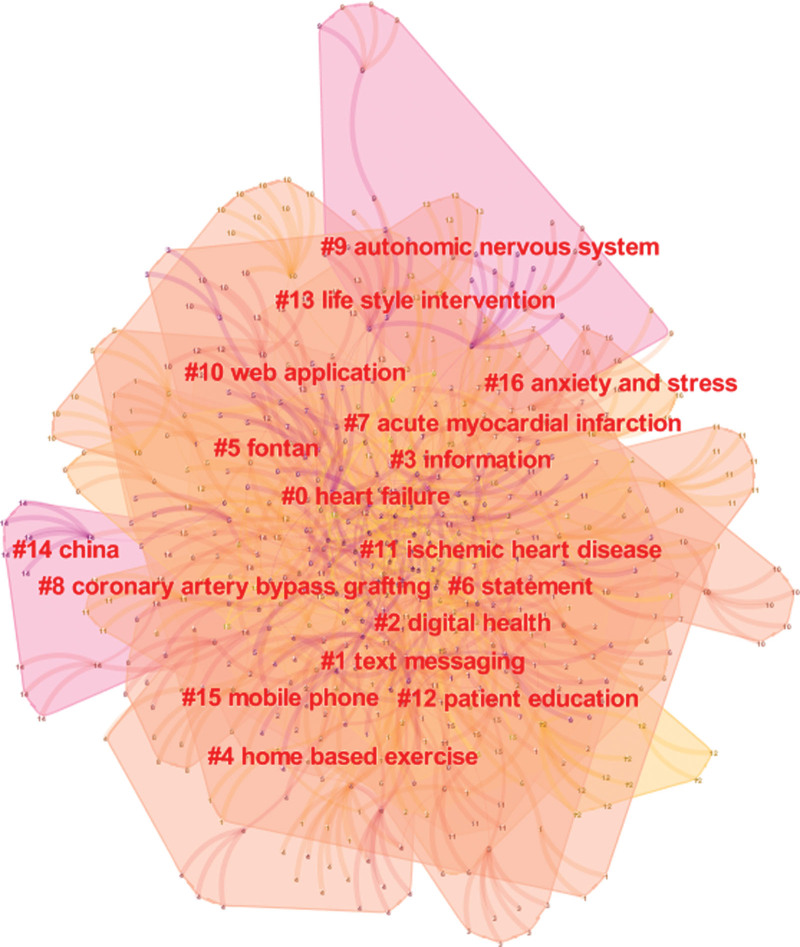
Keywords cluster analysis co-occurrence map.

As shown in Figure 9, keyword clustering describes the internal knowledge structure of a certain research field and classifies its domain.^[28]^ The clusters obtained after the analysis mainly included heart failure, text messaging, digital health, information, home based exercise, fontan, etc. The mean silhouette is usually used for evaluating the clusters. In general, a silhouette value over 0.7 means the cluster is high in efficiency and convincing; if it is above 0.5, the cluster is generally considered reasonable. Eventually, we obtained 23 clusters, and the silhouette value for each cluster was over 0.7, suggesting that the results were reliable and meaningful.

### 3.7. Bibliometric analysis of keywords with citation bursts

**Figure 10.**
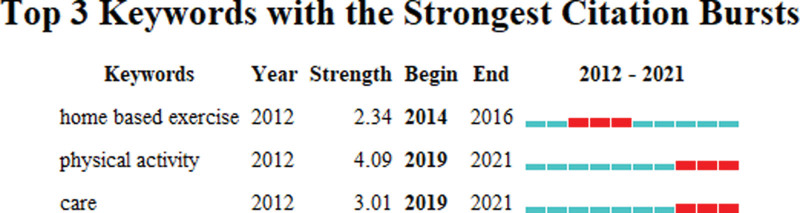
Top 3 keywords with the strongest citation bursts.

Figure 10 showed the top 3 keywords with the strongest citation bursts. The blue line represents the time interval, while the red line indicates the time period in which a keyword was found to have a burst.^[29]^ Keywords with citation bursts first appeared in 2014 (home based exercise), along with the strongest keyword (physical activity). The most recent keyword with citation bursts appeared in 2019 (physical activity, care) and continue to 2021.

## 4. Discussion

### 4.1. General information

A total of 296 articles were retrieved from the Web of Science database from January 2012 to December 2021 on the topic of home-based cardiac rehabilitation; these articles related to home-based cardiac rehabilitation were published by 1503 authors, by 626 institutions, and in 49 countries/regions. The number and trend of references published each year reflect the development speed and progress of a certain research field (Fig. 2).^[30]^ The number of publications has shown a trend of steady annual growth in the last 10 years, and the number of publications has increased by more than 4 times from 2012 to 2021. As shown in Table 4, RALPH MADDISON has published the most articles and his research in the field of home-based cardiac rehabilitation is relatively early. Table 2 and Figure 5 show that the US had the largest number of articles published in this field, with a total of 54 articles. In addition, this means that the US is the largest contributor to home cardiac rehabilitation research. This was followed by Australia, England, Canada, and Canada.^[24,31,32]^ Six countries, the Switzerland, England, Scotland, US, Australia, and Netherlands, present a high median centrality (≥0.1), which is considered a critical turning point leading to transformative discoveries. In addition, all of the top ten countries except China are developed countries. In recent years, China has ranked fifth in the number of published articles, making a great contribution to this field, possibly because the number of published articles and research results are related to national researchers and financial support. Eight of the ten institutions with the highest number of publications were universities, indicating that research related to home-based cardiac rehabilitation is concentrated in universities. The University of Sydney, University of York, University of Auckland, University of Exeter, and Institute of Cardiology (Mexico) published the most articles and also actively cooperated with each other; thus, they all have made significant contributions to the field of home-based cardiac rehabilitation.

### 4.2. Hot issues in HBCR research

Keyword co-occurrence and emergent item analysis can determine hot topics in a specific field in different periods and cluster keywords provided by authors in data sets. From the keywords, clusters, and keywords with citation bursts, it is clear that the main topics of interest in this field include the application of HBCR in different disease types; the application of different exercise programs in HBCR; and the application of digital health in HBCR.

#### 4.2.1. Actively explore the applicable population for home cardiac rehabilitation and expand the coverage of cardiovascular secondary prevention.

In order to give full play to the role of home-based cardiac rehabilitation in cardiovascular secondary prevention, it is necessary to actively explore the application of home-based cardiac rehabilitation in more patient populations. According to the high frequency keywords “myocardial infarction,” “heart failure,” “coronary heart disease,” it can be seen that myocardial infarction, coronary heart disease, and heart failure are among the most popular types of diseases studied in home-based cardiac rehabilitation. A large number of long-term studies have confirmed that the application of home-based cardiac rehabilitation in the above diseases can improve patients’ exercise capacity and quality of life with the same and better results than center-based cardiac rehabilitation than conventional care.^[33,34]^ The emergence of cluster #5 fontan suggests that some researchers have begun to focus on cardiovascular disease populations other than coronary heart disease and heart failure. Some studies suggest that home-based cardiac rehabilitation is equally effective in patients undergoing percutaneous coronary intervention, coronary artery bypass grafting, atrial fibrillation, heart transplantation, and valve repair.^[35–39]^ The European Society for Cardiac Prevention has called for future trials in additional patient populations such as pulmonary hypertension and precordial disease.^[40]^ In addition, previous studies have focused on middle-aged male patients, while women and older adults are underrepresented in the home-based cardiac rehabilitation trial population.^[41]^ Female patients are more likely to suffer from psychosocial problems such as anxiety and depression,^[42]^ and elderly patients generally suffer from cognitive and physical decline,^[43]^ which limit the application of home-based cardiac rehabilitation in female and elderly populations. In the future, the factors influencing the participation of women and elderly groups in home-based cardiac rehabilitation should be explored in depth, and individualized home-based cardiac rehabilitation programs should be developed based on the physical and psychological characteristics of women and elderly groups. In addition, the implementation of home-based cardiac rehabilitation is currently concentrated in developed countries such as Europe and the United States, while low-income countries and rural areas have difficulty in implementing home-based cardiac rehabilitation due to financial constraints and lack of infrastructure and a sound cardiac rehabilitation system.^[44]^ Future research should focus more on the cost and cost-effectiveness of home-based cardiac rehabilitation, and seek effective measures to lower the threshold of home-based cardiac rehabilitation from an economic perspective to meet the needs of cardiovascular patients in low-income regions or countries for home-based cardiac rehabilitation.

#### 4.2.2. Develop an appropriate home-based cardiac rehabilitation exercise program to improve patient compliance.

Exercise is a core component of home-based cardiac rehabilitation. This means that adherence to a home-based cardiac rehabilitation program is essentially the same as adherence to a home exercise program. Exercise-based cardiac rehabilitation has been shown to be the third most cost-effective intervention for reducing cardiovascular disease mortality, after aspirin and beta-blockers.^[45]^ Current international guidelines recommend an exercise regimen for home-based cardiac rehabilitation that includes mainly aerobic and resistance exercise, but there is no consensus on recommendations for exercise intensity due to national and regional differences. The American and European Cardiac Rehabilitation Associations recommend that the intensity of exercise should be moderate to vigorous.^[46,47]^ In the past few years, high-intensity intermittent exercise has received more attention and researchers have tried to apply it in home-based cardiac rehabilitation programs. The greatest advantage of high-intensity intermittent exercise is that it can significantly increase the patient’s peak oxygen uptake and thus improve cardiopulmonary function.^[47]^ However, more reliable evidence is needed to support the safety of high-intensity intermittent exercise in the home setting without medical supervision. In Australia and the United Kingdom, aerobic exercise of light to moderate intensity is recommended, and this recommendation is also recommended by the World Health Organization (WHO) for developing countries. In addition, in addition to common forms of exercise such as jogging, cycling, and swimming, researchers in each country or region could actively try to incorporate exercise programs with local traditions or characteristics into home exercise programs, which would theoretically increase patient interest and acceptance of home-based cardiac rehabilitation programs to some extent, which may be a way to obtain better participation and completion rates. For example, Chinese taijiquan^[48]^ and Indian yoga^[49]^ have been used in home-based cardiac rehabilitation programs with good results.

#### 4.2.3. In-depth integration of electronic information technology such as the internet, the development of home cardiac remote rehabilitation.

With the rapid development of electronic information technology such as the Internet, the application of technologies such as wearable devices (heart rate monitors, accelerometers) and telecommunication (video telephony) has improved the system composition of home-based cardiac rehabilitation. Relying on electronic information technology, it is possible to monitor the patient’s physical status in real time and provide virtual face-to-face guidance, improving the safety of home-based cardiac rehabilitation.^[50]^ The use of mobile applications has been shown to maintain patients’ physical activity levels over time,^[51]^ which means that the long-term benefits of home-based cardiac rehabilitation for patients are expected to be confirmed by the introduction of electronic information technology. The global pandemic of COVID-19 in recent years has severely limited CBCR, forcing nearly half of all patients to discontinue cardiac rehabilitation,^[52]^ and the need to maintain social distance has led to the rapid development of home-based cardiac tele-rehabilitation as the best alternative for both healthcare providers and patients, which has in part contributed to the rapid development of home-based cardiac tele-rehabilitation. It is important to note that despite the high availability and acceptance of home-based cardiac tele-rehabilitation, some external variables such as component quality, system quality, intrinsic factors and facilitation, need to be further refined. The European Society for Cardiac Prevention^[44]^ more clearly identifies several major barriers to the implementation of tele-rehabilitation programs: different digital literacy of patients and healthcare professionals; cost reimbursement; electronic medical record integration; lack of face-to-face interaction; data security and user privacy; and lack of legislation, in addition to the special attention that should be paid to remote clinical assessment and regulatory issues. The above-mentioned issues are not only the focus and difficulty of future research, but also the key to the popularity of home cardiac tele-rehabilitation.

## 5. Strengths and limitations

To the best of our knowledge, this is the first time CiteSpace has been used to conduct bibliometric analysis and for the visual display of HBCR in terms of hot spots, co-cited literature, and collaborations between countries, institutions and authors. The data downloaded from the WoSCC database covered the vast majority of articles in the field of HBCR research, and the data analysis was relatively objective and comprehensive, which clarified the past and present situation of HBCR and predicted future research frontiers. However, this study also had some limitations. Firstly, the limitations of CiteSpace meant that we only downloaded literature analysis from WoSCC; therefore, our data might not represent all the available literature. Secondly, our study defined certain keywords that may lead to data reduction. Thirdly, our analysis only included articles in English, which made the analysis incomplete to some extent. Finally, because of the existence of multiple synonyms, there might be some overlap between different categories of content in the keyword clustering.

## 6. Conclusion

Research in home-based cardiac rehabilitation has been of high interest to researchers, and research in this field is concentrated in developed countries such as the United States and the United Kingdom, where universities are the main research institutions and there are strong national/regional and institutional collaborations. Currently, research topics and hotspots in this field are focused on disease types, exercise protocols, and digital tele-rehabilitation.

## Author contributions

**Conceptualization:** Jingyu Liu, Lingyu Wang.

**Data curation:** Jingyu Liu.

**Formal analysis:** Jingyu Liu.

**Investigation:** Jingyu Liu.

**Methodology:** Jingyu Liu, Lingyu Wang.

**Software:** Lingsha Wu, Jing Zhang.

**Visualization:** Lingsha Wu, Jing Zhang.

**Writing – original draft:** Jingyu Liu, Lingyu Wang.

**Writing – review & editing:** Haiyan Fang, Xiang Wang.

## Supplementary Material


